# High-Quality Sequencing, Assembly, and Annotation of the Streptomyces griseofuscus DSM 40191 Genome

**DOI:** 10.1128/MRA.01100-20

**Published:** 2020-11-19

**Authors:** Tetiana Gren, Tue S. Jørgensen, Christopher M. Whitford, Tilmann Weber

**Affiliations:** a The Novo Nordisk Foundation Center for Biosustainability, Technical University of Denmark, Kgs. Lyngby, Denmark; University of Maryland School of Medicine

## Abstract

Here, we report the sequencing, assembly, and annotation of the genome of Streptomyces griseofuscus DSM 40191. The genome of *S. griseofuscus* was sequenced using PacBio and Illumina technologies. It consists of a linear 8,721,740-bp chromosome and three plasmids, pSGRIFU1 (220 kb), pSGRIFU2 (88 kb), and pSGRIFU3 (86 kb).

## ANNOUNCEMENT

Streptomyces griseofuscus DSM 40191 (NRRL B-5429) was originally isolated from a soil sample collected near Dake Hot Spring, Fukushima Prefecture, Japan (1), as a producer of the antibiotics bundlin A and B (lankacidins C and A) and later submitted to the DSMZ collection. We believe that this strain is an interesting producer of various secondary metabolites and a promising heterologous expression host. Its current RefSeq genome assembly (accession number GCF_000718315.1) is fragmented into 244 contigs and is not suited for genome mining (2). The strain was grown in yeast extract-malt extract (YEME) medium according to reference 3, and genomic DNA was purified using the Genomic Tip 100 kit (Qiagen, Venlo, Netherlands). An Illumina (San Diego, CA) whole-genome sequencing (WGS) library was constructed using the KAPA (St. Louis, MO, USA) HYPRplus kit, and 3,535,535 read clusters were generated on an Illumina MiSeq sequencer with a 2× 150-nucleotide (nt) kit. Macrogen, Inc. (Seoul, South Korea), generated the PacBio (Menlo Park, CA, USA) RS II data (191,463 subreads; *N*_50_, 15,731 nt) using the 8PAC V3, DNA polymerase binding kit P6, and 2 single-molecule real-time (SMRT) cells; a g-TUBE (Covaris, Inc., Woburn, MA, USA) was used for DNA shearing followed by BluePippin size selection (Sage Science, MA, USA). Default software parameters were used except where otherwise noted. The Illumina data were adapter trimmed using AdapterRemoval v2 (v.2.2.2) (4) with the switches “–trimns –trimqualities.” The PacBio subreads were assembled with Flye (v.2.4.1-geb89c9e) (5) with the switches “–genome-size 8m –iterations 5.” Then, the assembly was polished with the polishing module of Unicycler using the Illumina data (Unicycler v. 0.4.8-beta) (6). The inverted repeats, which are typically found at the ends of the linear *Streptomyces* chromosomes, were manually added according to the assembly graph, and the genome was polished again. We confirmed that the inverted repeat ends of the chromosome were correctly attached by identifying reads overlapping the junctions using minimap2 (v. 2.16-r922) (7), Bowtie 2 (v.2.3.5) (8), and Artemis (v.18.0.2) (9). The telomeric sequence from the related strain Streptomyces rochei 7434AN4 (accession number AB905441.1) was found within the inverted repeat but not at the absolute terminus. The coverage of PacBio data was found to be uniform and ca. 150× in the chromosome. The assembled genome was annotated using Prokka (v.1.14.0) with the default databases as well as the PFAM-A (v.32.0) (10) database and six actinobacterial genomes as reference data sets (NC_003155.5, NC_004719.1, NC_010572.1, NC_008596.1, NC_014318.1, NC_003888.3, NC_003903.1, NC_003904.1, NC_021985.1, NC_022001.1, and NC_021986.1). RNAmmer (v.1.2) (11) was used for rRNA predictions. The polished genome of *S. griseofuscus* consists of a chromosome of 8,721,740 bp with inverted repeat ends of ca. 84 kb and the following three plasmids: pSGRIFU1 (220 kb), pSGRIFU2 (88 kb), and pSGRIFU3 (86 kb). The assembly graph reveals the architecture of the linear genome with inverted repeat ends and two linear plasmids (pSGRIFU1 and pSGRIFU2) and a third (pSGRIFU3) that is highly repetitive and has a complex assembly path that is not resolved. The average GC content of the genome is 71.63%. Of the 8,114 coding DNA sequences (CDSs), 5,893 (73%) were functionally annotated. A total of 292/292 (100%) BUSCO (v.4.0.5) (12) actinobacterial core genes (actinobacteria_class_odb10 ortholog set) were found in single copy. Comparing the final assembly to the existing draft RefSeq version (GCF_000718315.1) of *S. griseofuscus*, the main difference is the continuity, with the final assembly being complete. Furthermore, mapping the existing draft RefSeq version (GCF_000718315.1) to the finished version and calculating the differences between them with minimap2 (with the switch “-N 1000”) and SAMtools (v.1.7, using htslib 1.7–2) (13), we found a total of 186,462 nt in the present assembly which are not covered by the draft version (GCF_000718315.1), including the majority of the inverted repeat chromosome ends and several gaps between the contigs, as well as 44 single nucleotide variations and six insertions and deletions.

### Data availability.

All data are available as BioProject PRJNA622435. Raw reads are deposited at SRA with accession numbers SRR11462380 (Illumina) and SRR11462379 (PacBio). The GenBank accession numbers are CP051006, CP051007, CP051008, and CP051009.
